# Research priorities for European paediatric emergency medicine

**DOI:** 10.1136/archdischild-2019-316918

**Published:** 2019-04-25

**Authors:** Silvia Bressan, Luigi Titomanlio, Borja Gomez, Santiago Mintegi, Alain Gervaix, Niccolo Parri, Liviana Da Dalt, Henriette A Moll, Yehezkel Waisman, Ian K Maconochie, Rianne Oostenbrink

**Affiliations:** 1 Department of Pediatrics, University of Padova, Padova, Italy; 2 Pediatric Emergency Department, Hopital Universitaire Robert-Debre, Paris, France; 3 Inserm U1141, Paris, France; 4 Pediatric Emergency Department, Hospital Universitario Cruces, Barakaldo, País Vasco, Spain; 5 Pediatrics, University of Geneva, Geneva, Switzerland; 6 Emergency Department & Trauma Center, Ospedale Pediatrico Meyer Firenze, Florence, Italy; 7 General Paediatrics, Erasmus MC-Sophia Children’s Hospital, Rotterdam, The Netherlands; 8 Pediatric Emergency Department, Schneider Children’s Medical Center, Day Care Unit, Petah Tikva, Israel; 9 Paediatric Emergency Department, Imperial College Hospital NHS Healthcare Trust, London, UK

**Keywords:** health services research, epidemiology

## Abstract

**Objective:**

Research in European Paediatric Emergency Medicine (REPEM) network is a collaborative group of 69 paediatric emergency medicine (PEM) physicians from 20 countries in Europe, initiated in 2006. To further improve paediatric emergency care in Europe, the aim of this study was to define research priorities for PEM in Europe to guide the development of future research projects.

**Design and Setting:**

We carried out an online survey in a modified three-stage Delphi study. Eligible participants were members of the REPEM network. In stage 1, the REPEM steering committee prepared a list of research topics. In stage 2, REPEM members rated on a 6-point scale research topics and they could add research topics and comment on the list for further refinement. Stage 3 included further prioritisation using the Hanlon Process of Prioritisation (HPP) to give more emphasis to the feasibility of a research topic.

**Results:**

Based on 52 respondents (response rates per stage varying from 41% to 57%), we identified the conditions ‘fever’, ‘sepsis’ and ‘respiratory infections’, and the processes/interventions ‘biomarkers’, ‘risk stratification’ and ‘practice variation’ as common themes of research interest. The HPP identified highest priority for 4 of the 5 highest prioritised items by the Delphi process, incorporating prevalence and severity of each condition and feasibility of undertaking such research.

**Conclusions:**

While the high diversity in emergency department (ED) populations, cultures, healthcare systems and healthcare delivery in European PEM prompts to focus on practice variation of ED conditions, our defined research priority list will help guide further collaborative research efforts within the REPEM network to improve PEM care in Europe.

**What is already known on this topic?**

- Paediatric emergency medicine (PEM) has been a well-recognised subspecialty for decades in Canada, the USA and Australia.
- Research in European Paediatric Emergency Medicine (REPEM) network is faced with unique challenges related to its high diversity of countries, languages and healthcare systems.

**What this study adds?**

- REPEM members identified common research priorities on the following themes: ‘fever’, ‘sepsis’, ‘respiratory infections’, ‘biomarkers’, ‘risk stratification’ and ‘practice variation’.
- The high diversity in ED populations, cultures, healthcare systems and healthcare delivery in European PEM prompts to focus on practice variation of ED conditions.
- Large multicentre collaborations such as REPEM are nowadays essential to include large population samples to study rare but high-impact conditions, such as sepsis, and risk stratification in European PEM.

## Introduction

Paediatric emergency medicine (PEM) has been a well-recognised subspecialty for decades in Canada, the USA and Australia.^1–3^ PEM physicians own a unique skillset to provide optimal appropriate and efficient care to acutely ill and injured children in the dynamic, multitasking and often overcrowded environment of the emergency department (ED). In this setting, the few children with serious conditions blend within the great majority of patients presenting with self-limiting illnesses. Despite the lack of official recognition of its own identity and formal training requirements in most European countries, except for the UK, Switzerland, Turkey and Israel, PEM has been practised in many countries in Europe, leading to heterogeneous quality of care of children presenting to European paediatric or mixed EDs.^4 5^

Following worldwide initiatives,^2 3 6 7^ the Research in European Paediatric Emergency Medicine (REPEM) network was founded in 2006 to improve emergency care for children in Europe,^4 8 9^ consisting of 69 partners from 20 European countries. The UK and Ireland have founded their own research network^6^ but collaborate with REPEM in some research projects. Compared with existing (multi)national networks of English-speaking countries, REPEM is faced with unique challenges related to its high diversity in ED populations, cultures and diversity in delivered health and healthcare systems. Similar to other research networks,^2 3 7 10 11^ we need to establish our research agenda relevant to PEM in Europe to guide the development of future research projects, which should not be exclusively based on members’ interests and prior work in a specific research field.

Therefore, the aim of this study was to define research priorities in the area of PEM for a collaborative network in Europe.

## Methods

### Study design

**Figure 1:**
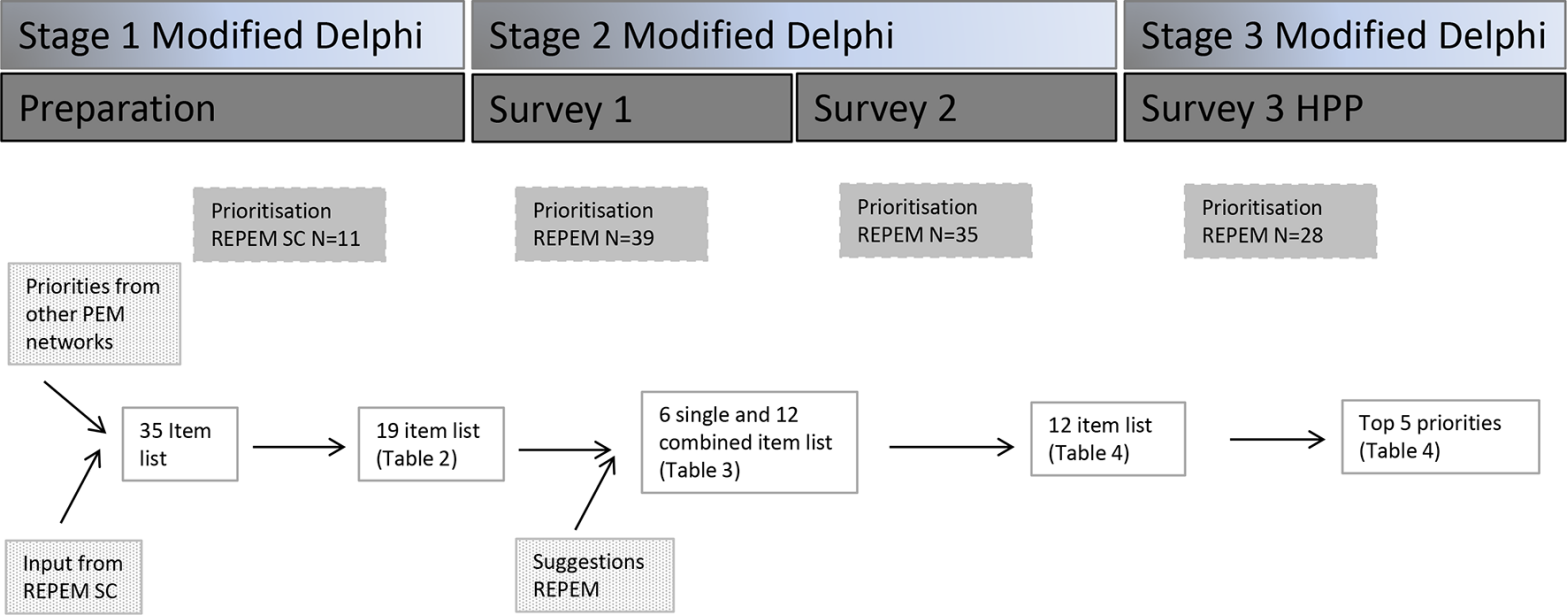
Flow chart of the study. HPP, Hanlon Process of Prioritisation; PEM, paediatric emergency medicine; REPEM, Research in European Paediatric Emergency Medicine; SC, steering committee.

We carried out an online survey in a modified three-stage Delphi study (figure 1).^12^ Other research networks have conducted similar studies to systematically determine research priorities using consensus methodology.^2 3 7 10^ Eligible participants were invited from the REPEM network, consisting of 69 members.

### Modified Delphi stage 1 preparation

This stage aimed to define the list of research topics to be ranked further by REPEM members in the Delphi process.

The REPEM steering committee (SC, n=11 members) was asked to review priority items from previous publications of other PEM networks (PERC, PERUKI, PERN, PREDICT)^2 3 7 10^ and to add items to this list if considered important from the European perspective. To this composed list SC members then assigned priorities by scaling items from 1 (low) to 6 (high priority). Items ranked 4 or higher by >6 out of 11 SC members selected for the final list used in stage 2.

### Modified Delphi stage 2

Stage 2 consisted of two online surveys. All REPEM members were asked to prioritise each item, on a scale of 1–6. They could add comments and suggest additional (missing) topics. Based on the survey’s results and respondents’ comments, the list of topics was refined and reduced for the second survey. Suggested items were added. Items with two-third or more respondents ranking them 4 or lower on the priority scale were removed.

### Modified Delphi stage 3: Hanlon Process of Prioritisation

In a modified Hanlon Process of Prioritisation (HPP),^2 13^ we weighted prevalence and seriousness of the condition and feasibility of conducting research projects on that condition to prioritise health condition. An HPP score was calculated for each item using the mean scores for each of the three domains (A, B, C) as follows: HPP=(A+2B)×C. This process was carried out by a third online survey.

### Analysis

In each stage, we computed cumulative percentages for the priority levels for each item and mean priority scores with SD.

## Results

### Participants’ responses to surveys

All 11 SC members participated in the preparation phase and contributed to modified Delphi stage 2 and 3. In total, 52 of the 69 invitees participated in one of the Delphi stages with response rates varying from 41% to 57% (table 1). Participants were all PEM specialists, practising in tertiary EDs.

**Table 1:** Number of REPEM respondents per country and per survey phase

	Stage 2		Stage 3	Total
	Survey 1	Survey 2	Survey 3, HPP	
Invited REPEM members	69	69	69	69
Austria	1	1	1	1
Belgium	1	1		1
France	4	1	3	4
Germany	1	1		1
Hungary	2	1	2	3
Israel	1	2	1	2
Italy	5	4	2	6
Latvia	1	1	1	1
Lithuania	1			1
Malta		1		1
The Netherlands	3	3	1	3
Portugal		1	1	1
Spain	6	6	5	7
Sweden		2		2
Switzerland	6	3	5	7
Turkey	4	5	3	6
UK	3	2	3	4
All	39 (57%)	35 (51%)	28 (41%)	52 (75%)

HPP, Hanlon prioritisation process; REPEM, Research in European Paediatric Emergency Medicine.

### Modified Delphi stage 1 (preparation)

In addition to other PEM-networks research priority exercises, the SC suggested ‘child abuse’, ‘care of refugee children’, ‘ultrasound’, ‘simulation’, ‘implementation’, ‘patient-reported outcomes’ and ‘pathways of ED conditions’ as additional priority topics. Items ‘new technologies’, ‘risk stratification’, ‘antibiotic stewardship’ and ‘fluid therapy in gastroenteritis’ were rephrased from other PEM-networks prioritised items. Stage 1 ended with a list of 35 topics, on areas infectious diseases, the critically ill patient, paediatric trauma, neurology, diagnostic procedures, therapy and items of organisation of care and quality of care. By prioritisation, 19 items were selected by the SC for the further Delphi stages (table 2).

**Table 2:** Priority ranking Delphi stage 2, survey 1 (n=39 respondents)

Type	Topic	Mean priority (SD)	Lowest priority assigned	Highest priority assigned	% prioritising 5 or higher
C	Sepsis	4.97 (1.04)	2	6	69.2
C	Fever	4.95 (1.15)	2	6	69.2
C	Respiratory illness	4.87 (1.06)	2	6	71.8
C	Paediatric cardiopulmonary arrest	4.67 (1.36)	2	6	56.4
C	Child abuse	4.36 (1.04)	2	6	46.2
C	Care for refugee children	3.87 (1.32)	1	6	35.9
I	Risk stratification	5.08 (0.98)	2	6	74.4
I	Biomarkers	5.05 (0.83)	3	6	79.5
I	New PED technologies	5.05 (0.97)	3	6	76.9
P	Knowledge translation/implementation	4.69 (1.03)	2	6	61.5
P	Antibiotic stewardship	4.69 (1.26)	1	6	59.0
P	Pathways of PED conditions	4.59 (1.04)	2	6	61.5
I	Triage	4.59 (1.19)	2	6	61.5
I	Ultrasonography	4.54 (1.17)	1	6	61.5
P	Simulation	4.54 (1.32)	2	6	51.3
I	Procedural sedation and analgesia	4.51 (1.21)	1	6	59.0
P	PED organisation	4.44 (1.21)	2	6	46.2
P	Patient-reported outcomes	4.33 (1.24)	1	6	41.0
I	PEWS	4.23 (1.20)	2	6	46.2

C, condition, I, intervention; P, process; PED, paediatric emergency department; PEWS, paediatric early warning score.

### Modified Delphi stage 2 (two surveys)

In the first survey, ‘refugee children’ achieved lowest priority (3.87); ‘risk stratification’ highest (5.08) (table 2). Items selected included ‘fever’, ‘sepsis’, ‘respiratory illness’, ‘biomarkers’, ‘risk stratification’, ‘new technologies’. The majority ranked ‘child abuse', care of refugee children’, ‘paediatric early warning score’, ‘ED organisation’, ‘patient-reported outcomes’ to scores 4 or lower. Suggestions included ‘malpractice’, ‘health services research’, ‘trauma’ (management of major trauma and minor head trauma) and ‘poisoning’. The latter two were suggested during the preparation phase, but did not achieve sufficient priority then. During refinement, processes/interventions were specified for specific conditions, for example, ‘biomarkers for fever’ and ‘risk stratification in trauma’.

Survey 2 included five conditions, three processes and two interventions. Next, we composed combined items for conditions and processes/interventions, and open questions to suggest new technologies for these conditions. ‘Biomarkers in trauma’ achieved lowest priority scores (3.91); ‘risk stratification in sepsis’ highest (5.11) (table 3). Although suggested in survey 1, ‘poisoning’ did not achieve sufficient priority and was together with ‘security’ and ‘access to healthcare’ removed for the next prioritisation round.

**Table 3:** Priority ranking Delphi stage 2, survey 2 (n=35 respondents)

Type	Item	Mean priority (SD)	Lowest priority assigned	Highest priority assigned	% prioritising 5 or higher
	***Items new suggested in/adapted after Delphi stage 1***				
C	Trauma	4.60 (1.29)	1	6	57.1
C	Poisoning	4.06 (1.06)	2	6	34.3
P	Health services research	3.89 (0.96)	2	6	22.9
P	Safety/security	4.14 (1.33)	1	6	42.9
P	Practice variation	4.51 (1.14)	2	6	60.0
I	Risk stratification	4.54 (1.17)	2	6	60.0
	***Combination of conditions and process/intervention***				
*P*	*Practice variation*				
	in sepsis	5.03 (0.92)	3	6	71.4
	in fever	4.66 (1.08)	2	6	57.1
	in RTI	4.60 (0.91)	2	6	51.4
	in trauma	4.37 (1.00)	2	6	42.9
*I*	*Risk stratification*				
	in sepsis	5.11 (.99)	3	6	71.4
	in fever	4.94 (1.00)	2	6	74.3
	in RTI	4.5 (1.04)	2	6	51.4
	in trauma	4.29 (1.32)	1	6	32.9
*I*	*Biomarkers*				
	in sepsis	5.09 (0.92)	3	6	74.3
	in fever	5.06 (1.06)	2	6	74.3
	in RTI	4.54 (1.20)	2	6	65.7
	in trauma	3.91 (1.40)	1	6	34.3

C, condition; I, intervention; P, process; RTI, respiratory tract infections.

Suggested new topics during the second survey included ‘standardised pathways of care’, ‘external validations for clinical outcomes’, ‘focus on young infants’, ‘e-health’ and ‘rapid tests for fever’. For trauma, suggested new technologies included ‘devices/guidance for intubation in the ED’, ‘CT, MRI and ultrasound in the ED’; other suggested items were use of ‘tranexamic acid’ and ‘prognosis’.

### Modified Delphi stage 3 (HPP)

The HPP identified highest priority for four of the five highest prioritised items by the Delphi (table 4). Feasibility concerns for ‘fever’ included the diversity of conditions covered by the broad term ‘fever’, and some members questioned its importance given the high amount of data available in literature. For ‘sepsis’ members commented on the need for definitions, and the low feasibility to detect unfavourable outcomes. Research in ‘respiratory infections’ was considered important given the high prevalence, but subject to the absence of a clear definition of ‘type and origin’. ‘Trauma’ was reported to be an under-researched area compared with other ED conditions, but hampered by low frequency of major trauma, and high diversity of trauma types. In addition, not all paediatric ED centres in Europe manage trauma.

**Table 4:** Delphi stage 3, HPP ranking of research topics in PEM (n=28 respondents)

HPP rank	Item	Mean priority (SD)	Delphi rank
1 (167)	Biomarkers in sepsis	5.09 (0.92)	2
2 (162)	Risk stratification in sepsis	5.11 (.99)	1
3 (150)	Practice variation in sepsis	5.03 (0.92)	4
4 (147)	Practice variation in fever	4.66 (1.08)	6
5 (146)	Biomarkers in fever	5.06 (1.06)	3
6 (144)	Practice variation in RTI	4.60 (0.91)	7
7 (141)	Risk stratification in fever	4.94 (1.00)	5
8 (140)	Risk stratification in RTI	4.5 (1.04)	9
9 (133)	Biomarkers in RTI	4.54 (1.20)	8
10 (127)	Practice variation in trauma	4.37 (1.00)	10
11 (124)	Risk stratification in trauma	4.29 (1.32)	11
NA	Biomarkers in trauma	3.91 (1.40)	12

HPP, Hanlon prioritisation process; NA, not available; PEM, paediatric emergency medicine.

## Discussion

In a modified three-stage Delphi among REPEM members, we identified common themes of research interest: ‘fever’, ‘sepsis’ and ‘respiratory infections’, ‘biomarkers’, ‘risk stratification’ and ‘practice variation’. Although not to be used as limitation, this list will help guide further collaborative research supported by our research network.

Incorporating prevalence, severity and feasibility of undertaking such research by using the HPP selected four of the top five prioritised items. The high priority to study practice variation of ED conditions may arise from the multinational, multicultural, multilanguage European landscape, with different healthcare systems and organisations. Correctly interpret variation in paediatric emergency care may elicit causative relations and contribute to inspiration for areas of improvement. Studying rare high-impact conditions as sepsis, and risk stratification for common conditions require large population samples to conduct adequately powered studies on low frequency-high stakes conditions and to ensure representativeness of European diversity in geography and culture for generalisable results. A critical mass can only be provided by large multicentre collaborations including a wide set of participant countries.^9^

Not all topics achieved sufficient high priority, due to larger respondents’ variability in prioritisations. Respondents may have prioritised differently based on availability, and feasibility for implementation in the respondents’ ED working environment. The priority ranking of ‘trauma’ was influenced by the diversity of the types of trauma and low prevalence of major trauma. So this research area needs to be better defined. ‘Poisoning’ has been studied already by REPEM members within the PERN collaboration.^4 5^ Topics ‘ultrasonography’, ‘triage’ and ‘procedural sedation’ with frequent scores of 5–6 (>60%) seem to be promising areas for successful collaborative research as well. Other topics, such as ‘implementation of knowledge’, ‘pathways of managing ED conditions’ and ‘in-house simulation’ reflect the need to efficiently implement knowledge into clinical ED practice. Dissemination and implementation of evidence-based guidelines for emergent conditions, customised to local practice is important,^11^ in particular considering the large number of children presenting to European EDs annually and the heterogeneous status of PEM in Europe. This will ensure provision of up-to-date optimal care, reduce variation in practice, improve patient outcome and experience of care as well as optimise resource utilisation.

Our results may deviate from other network priority exercises^2 3 7 10^ due to the survey itself or the intrinsic characteristics of REPEM. We did not define specific research questions, but merely identified research areas. Next, in contrast to other networks, REPEM members are all affiliated to tertiary paediatric emergency care settings with high research priority and do not cover the whole European area. This may have influenced the selected items, although our priority list includes the most common reasons for ED attendance in childhood, such as fever and respiratory infections, with dilemmas on risk stratification and diagnostic testing. Response rates were quite similar to surveys from other networks with participation of 75% of hospitals and 85% of countries involved in the REPEM network. Therefore, we think our results reflect the European research perspective in PEM. Other existing networks comprise English-speaking countries; the REPEM network consists of a high diversity of European countries, with their specific political, historical, cultural and socioeconomic traditions in the medical field, guidelines and healthcare systems. For example, Europe has a variable a priori risk of serious infections due to different vaccination strategies among countries. In addition, healthcare providers for emergent paediatric trauma range from community paediatricians (11% of countries) to paediatric surgeons (21% of countries); high-grade emergency care (meningitis/sepsis) is provided by either secondary, tertiary care or mixed.^14^ Recognising these differences is essential in developing evidence-based management strategies and support their implementation across Europe.

### Future of the REPEM network

Paediatrics ED presentations are responsible for a significant healthcare and economic burden because of non-urgent inappropriate visits to the ED, possible delayed diagnosis of serious diseases and balancing between overtesting and overtreatment of common benign conditions versus undertesting and undertreatment of rare serious conditions. In the European setting, there is the need for homogeneous epidemiological data on presenting paediatric emergent conditions and practice variation across European EDs, to best plan sample sizes for prospective studies and estimate the effects of implementing novel interventions. REPEM contributes to establishment of facilities (eg, common data collection and management system; harmonisation and standardisation of research procedures platform) and promote multidisciplinary collaboration, with primary focus on biomarkers for fever and sepsis, or other suggested new technologies by our study (imaging and intubation in the ED). To provide long-term sustainability of the network and advancement of research in PEM, we need investment in the capability of obtaining future funding for specific network research projects. This will lead to a long-lasting impact on clinical practice, to better patient care and a more cost-effective use of resources. The REPEM network has the task to contribute to knowledge development on best acute care practices and their implementation in routine clinical care to improve patient outcomes and experience of care incorporating the European diversity. In addition to clinical research experts in REPEM, it is essential to involve other key stakeholders (eg, experts on biostatistics, basic and translational research, effectiveness research and implementation science) to address the network’s challenges and objectives. We still need to promote inclusiveness of European countries' participation to PEM research to ensure a broad and diverse representation. Furthermore, we need to foster collaboration with patients and their families, for example, to include patient-centred and patient-reported outcomes in future study projects.^7 11^ This will ensure that the network’s research projects are relevant to both paediatric emergency care end-users and clinicians alike.
